# A remote group-mediated daylong physical activity intervention for older adults with chronic pain: Results of the MORPH-II randomized pilot trial

**DOI:** 10.3389/fdgth.2022.1040867

**Published:** 2022-11-02

**Authors:** Jason Fanning, Amber K. Brooks, Sherri Ford, Justin T. Robison, Megan B. Irby, W. Jack Rejeski

**Affiliations:** ^1^ Department of Health and Exercise Science, Wake Forest University, Winston Salem, NC, United States; ^2^ Department of Anesthesiology, Wake Forest School of Medicine, Winston Salem, NC, United States

**Keywords:** physical activity, sedentary behavior, pain, aging, technology

## Abstract

Chronic pain is a debilitating condition that affects many older adults who often have limited access to non-pharmacological pain management strategies. One potentially effective and novel lifestyle medicine for chronic pain involves increasing physical activity through frequent movement across the day, thereby also decreasing the presence of extended sedentary bouts. The MORPH-II pilot randomized controlled refinement trial iterated on the MORPH trial, which was a first-of-its-kind group-mediated daylong physical activity (DPA) intervention for older adults with chronic pain rooted in social cognitive and self-determination theories and supported by an mHealth toolset designed to foster social connection and awareness of physical activity patterns. MORPH-II was delivered fully remotely *via* videoconference software and supported by a technology kit comprising an iPad, activity monitor, and wireless weight scale. It was also implemented a refined coaching model designed to help participants better understand their own patterns of activity. A total of 44 participants were randomized to receive the 12-week group-mediated DPA intervention or to a low-contact control. Qualitative interviews suggest the program was well-received by participants and that participants developed an understanding of how patterns of physical activity related to their pain symptoms. Participants also highlighted several additional areas for refinement related to the coaching model and feedback provided within the mHealth app. Analyses of covariance, controlling for baseline values, revealed a small effect (*η*^2^ = 0.01) on pain intensity favoring the intervention condition, though both groups improved during the study period. There was a large effect favoring the intervention condition on ActivPAL-assessed average daily steps (*η*^2^ = 0.23) and postural shifts (*η*^2^ = 0.24). Control participants spent less time in short sedentary bouts (*η*^2^ = 0.09), and there was a small effect (*η*^2^ = 0.02) indicating intervention participants spent less time in extended sedentary bouts. Finally, relative to control, intervention participants demonstrated a moderate improvement in autonomy satisfaction (*η*^2^ = 0.05), relatedness frustration (*η*^2^ = 0.05), and competence frustration (*η*^2^ = 0.06), and a large magnitude improvement in competence satisfaction (*η*^2^ = 0.22). These findings indicate that the MORPH-II intervention was feasible and acceptable, and may positively impact steps, postural breaks, and several key domains of basic psychological needs detailed in self-determination theory.

## Introduction

Chronic pain is a common and burdensome chronic health condition that disproportionately affects older adults globally (1). It is debilitating, having a major impact on quality of life and independence, with many older adults not having access to effective non-opioid pain management strategies (2). A 2017 data brief from the Office of the Inspector General reported that one in three Medicare Part D (i.e., a United States federal health insurance program for those aged 65 or older with prescription drug coverage) beneficiaries received an opioid prescription in the previous year, and a half-million received “high amounts of opioids” (3). As noted in the 2021 annual report of the NIH *Helping to End Addiction Long-term* (HEAL) *Initiative* (4), evidence for long-term improvement in pain-related function with opioid medications is lacking, while higher medication-related symptoms (5) and increased harm are both well-documented with opioids (6). Rates of death from opioid overdose have increased dramatically in recent decades among older adults, rising from 0.90 per 100,000 persons aged 55 and older dying from an overdose in 1999 to 10.70 per 100,000 in 2019. There is substantial variation by race and ethnicity, with 40.03 overdose deaths per 100,000 among non-Hispanic Black men in 2019 (7). Chronic pain is also costly: in a 2012 report, Gaskin and Richard (8) found the incremental medical costs associated with chronic pain in adults and older adults totaled between $261–$300 billion annually. To that end, it is encouraging that the NIH—through the HEAL initiative—has highlighted the urgent need for behavioral programs targeting pain management (9).

**Figure 1:**
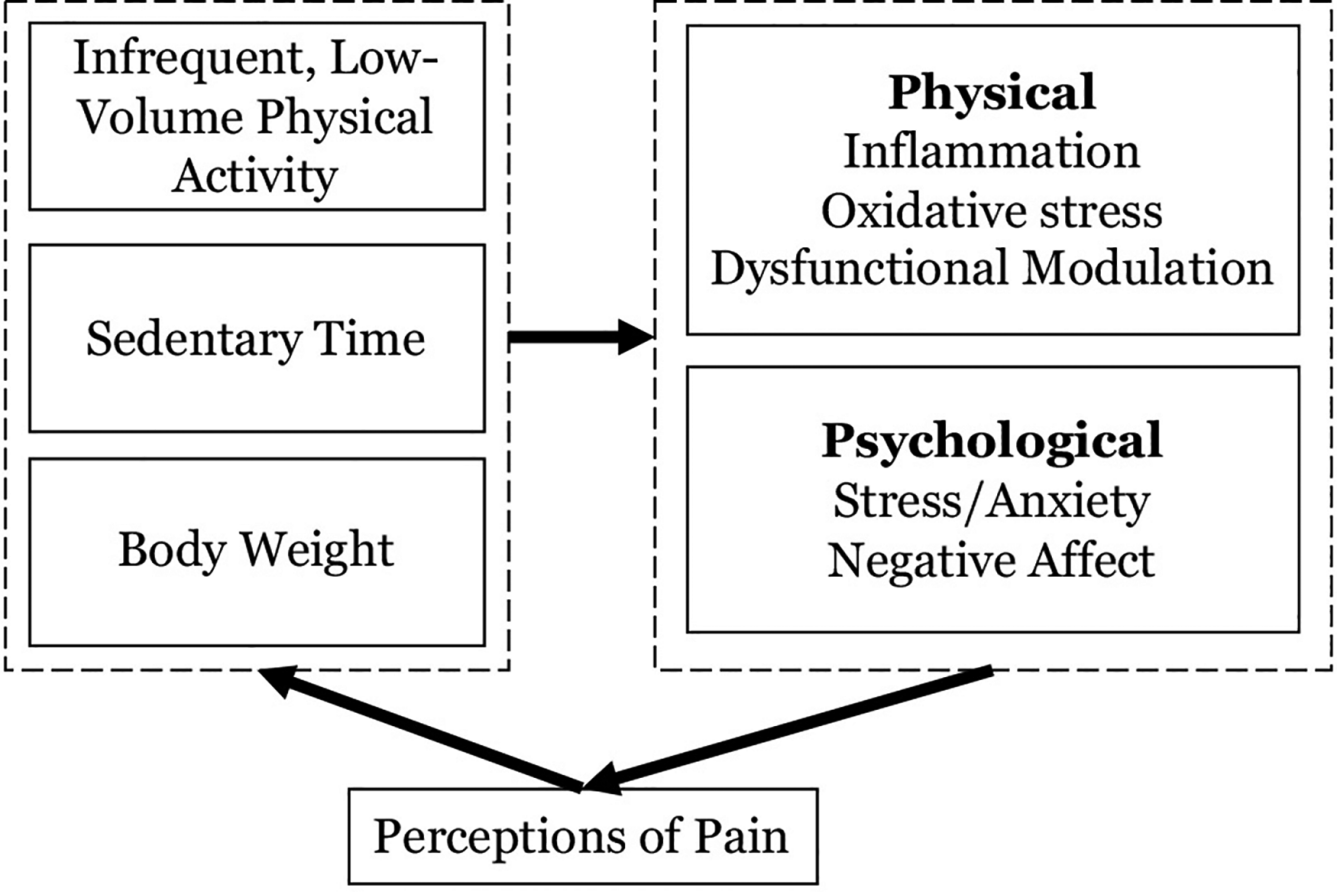
The cycle of movement dysregulation, excess body weight, and perceptions of pain.

Physical activity and sedentary behaviors affect and are affected by the experience of chronic pain. As described in the cyclical pathway depicted in Figure 1, the presence of prolonged periods of sitting paired with infrequent and/or low levels of light-intensity and moderate-to-vigorous intensity physical activity (LPA and MVPA respectively) are consistently associated with the experience of pain. Among late middle-aged and older adults in the Korean NHANES study, more time spent sitting—especially over 7 h—was associated with worse pain in the low back. These relationships were strongest among those engaging in the least leisure time physical activity (10, 11). In a meta-analysis of pain and sedentary behavior, Dzakpasu (10) found that more sedentary time was associated with greater pain in general, as well as low back and knee pain. In a longitudinal analysis of the relationships between accelerometer-measured sedentary time, MVPA, and pain among 199 women during and for 48-month following active breast cancer treatment, Doré and colleagues (12) found that more sedentary behavior was associated with increases in pain over time.

The tightly related lifestyle influences of sedentary and activity behaviors are thought to affect pain through a host of psychological and physiological mechanisms (select examples depicted in Figure 1). For instance, each of these inputs is associated with chronic inflammation and oxidative stress, which can induce sensitization of the central and peripheral nervous systems to pain (13). Moreover, experimental studies have demonstrated that MVPA and sedentary time are related to unique aspects of pain modulation (13). Emotion also plays an important role in acute and chronic pain, mediating the extent to which nociception (i.e., the neural encoding of a noxious stimulus) translates to perceptions of pain (14). Importantly, social connection plays a central role both in the experience of pain, as loneliness and pain operate bidirectionally (15, 16), and in the uptake and maintenance of challenging health behaviors (17, 18). Participation in regular physical activity—especially in a social context and when the activity is of moderate intensity in an intrinsically enjoyable format—and avoiding prolonged sitting is widely acknowledged as an effective medicine for addressing negative emotional states such as anxiety and negative affect (19). It is also notable that MVPA and sedentary behavior are interrelated and the relationships between these behaviors are affected by pain (20). The promotion of MVPA *via* structured exercise in older adults can contribute to compensatory behaviors, such as reduced lifestyle activities and increased sitting, and predispose a person to long-term weight gain (21). This phenomenon is likely to be exacerbated among those living with chronic pain who may harbor fear that exercise will exacerbate pain symptoms (22), which causes the anticipation of pain to become a strong barrier to sustaining highly effortful activities like structured exercise (20, 23).

Herein we present the findings of the *Mobile Intervention to Reduce Pain and Improve Health II* (MORPH-II) trial (24), the second in a series of iterative randomized controlled pilot trials with the aim of designing and establishing the feasibility and acceptability of a unique remote group-mediated behavioral intervention targeting the accumulation of physical activity throughout the day—an mHealth-supported “daylong physical activity” (DPA) protocol designed to increase stepping activity while indirectly disrupting sustained sitting behavior. The MORPH DPA intervention is designed within social cognitive (25, 26) and self-determination (21) theories, with the aim of enhancing self-efficacy and self-regulatory skills while supporting autonomy, competency, and relatedness which in-turn support long-term behavior change and well-being (17). The protocol manuscript (24) includes a detailed description on the model underlying MORPH-II.

The first iteration of MORPH, which included a focus on dietary weight loss, contributed to improvements in pain intensity, body weight, physical function, and several markers of quality of life (27, 28). However, there were also several important limitations requiring remediation prior to the conduct of a large, randomized trial. First, MORPH required participants to attend three in-person group meetings before transitioning to video conference delivery. This requirement limited the reach of the program to those willing to commute to the research center and participants expressed a desire to not change the meeting medium from in-person to remote delivery. Second, there was mixed uptake of the DPA program, such that some participants found the approach intuitive while others found it difficult to reconceptualize physical activity as anything other than traditional structured exercise. Participants who did not understand the DPA protocol also found little utility in the visual feedback provided in the MORPH “*Companion”* mHealth app (described further below). As a result, group-level changes in markers of physical activity and sedentary behavior were modest.

In the spirit of contemporary engineering-inspired intervention optimization frameworks (29, 30). MORPH-II is an iteration on the MORPH pilot trial with the aim of establishing the feasibility, acceptability, and initial impact of a refined MORPH intervention relative to a measurement control condition on pain, physical activity, sedentary breaks (i.e., postural transitions), and self-determinative mediators of behavior change and well-being. The two primary refinements include: (1) the addition of a pre-health student coaching model designed to provide low-cost individualized coaching to instill a deeper understanding of the DPA prescription and associated mHealth tools; and (2) a shift to fully remote delivery, including participant recruitment, testing, orientation, and conduct of the group-mediated intervention. Additionally, the MORPH-II educational content was reorganized to place a stronger emphasis on DPA relative to the first MORPH trial, which emphasized nutrition and weight loss. During the conduct of MORPH-II, feedback from participants and the intervention team was discussed as a group and refinements were made between waves as needed to allow for piloting these modifications. What follows is a description of the MORPH-II trial as well as both qualitative and quantitative data collected at the primary endpoint of the study (i.e., week 12).

## Methods

The institutional review board at Wake Forest University approved all study-related procedures, and the trial was registered at ClinicalTrials.gov (NCT04655001). Individuals who responded to recruitment efforts and passed a preliminary telephone screening received a copy of an informed consent document in the mail. They were asked to read the document in its entirety. A trained study staff member reviewed all key aspects of the consent document and study procedures verbally *via* telephone, provided opportunity for questions, confirmed understanding, and acquired verbal consent after all questions were answered. This was recorded by the study staff member. The MORPH-II trial was a 12-week two-arm randomized pilot trial, which was described in full in a protocol publication (24). Recruitment occurred in five waves between 2021 and 2022.

### Participant identification

Men and women from communities surrounding Charlotte, Durham, Greensboro, Raleigh, and Winston-Salem, North Carolina were recruited using digital and print newspaper advertisements, targeted postcard mailings, and targeted phone calls, email, or postal mail to individuals in databases of older adults interested in participating in research maintained by the Wake Forest Pepper Center.

### Inclusion and exclusion criteria

Eligible participants were late-middle-aged and older adults aged 55–85 years with obesity (body mass index of 30–45 kg/m^2^ based on self-reported height and weight, corrected *via* the Shields equation) (31), who were English-speaking, had self-reported pain in at least 1 of 3 areas (back, hip, or knees) on most days during the previous three months, low-active (i.e., engaging in less than 2 days/week of structured physical activity for at least 20 min), willing to be randomized to the MORPH-II intervention condition or to a measurement control condition and to complete study assessments, willing to use a study-provided iPad device, able to walk without a cane or walker, and with no contraindication for participation in exercise. Additionally, a secondary purpose of MORPH-II, which is not reported herein, was to pilot test an in-home short physical performance battery (32); thus, participants were required to have access to a smartphone to facilitate safety monitoring *via* videoconference during baseline testing. Participants were excluded if they were not weight stable as indicated by a self-reported loss or gain of at least 5% body weight in the previous 6 months, had fallen more than once in the previous year, had vision that could not be corrected sufficiently to view a smartphone or tablet screen, demonstrated impaired cognition as indicated by a modified telephone interview for cognitive status (33) score of less than 32, had uncontrolled hypertension, or had any of the following within the previous year: severe symptomatic heart disease, uncontrolled angina, stroke, osteoporosis, chronic respiratory disease requiring oxygen, neurological or hematological disease, or cancer requiring treatment except non-melanoma skin cancers. Participants were also excluded for regular use of growth hormones, oral steroids, or prescription osteoporosis medications, or if they received orthopedic surgery or joint replacement in the previous 6 months or planned to have such a surgery in the coming 6 months. Finally, participants were excluded if they were presently engaging in another research study targeting pain, physical activity, or weight loss, or if they participated in the first MORPH program. Eligible participants were randomized 1 : 1 into the MORPH intervention or a “measurement” control using permuted block randomization generated *via* a web-based tool. Baseline data were entered into a secure data management service operated by the Wake Forest Pepper Center, which concealed randomization assignment from assessment staff until all baseline data were entered and a randomization button was clicked.

Following randomization, all participants received their group-specific technology kit and then engaged in a one-on-one technology orientation meeting *via* phone or video conference. For those in the control condition, this meeting introduced the use of the Fitbit activity monitor and BodyTrace scale. For those in the MORPH condition the orientation covered key features of the Companion app, with an explanation of each piece of visual feedback, and the video conference software. These orientations were designed to enhance self-efficacy through mastery. Participants attempted to guide the study staff member through any technological interface, narrating aloud what they believed they saw. This process allowed participants to practice key interaction gestures while learning the skill of exploration. The pair then debriefed on all key aspects of the interface.

### Interventions

#### The measurement control

All participants received a Fitbit Inspire activity monitor and BodyTrace cellular scale to account for any effect on physical activity or body weight associated with receipt of these devices. Those in the measurement control condition were instructed on how to use these devices to monitor their activity behaviors and body weight and were asked to live their lives as normal for 24 weeks. During the 12-week intervention period that is the focus of this report, these participants received brief monthly telephone contacts wherein participants could ask questions related to the use of these devices, and the intent of these contacts was to bolster retention of control participants. Participants also completed testing once more at 24 weeks. This report is focused on the 12-week intervention period.

#### The MORPH intervention group

Those assigned to the MORPH intervention condition participated in weekly small group meetings led by a professional behavioral coach *via* Zoom. To facilitate participation in these sessions, participants in the MORPH intervention received the Companion app and technology kit after randomization. This included an iPad tablet computer with Wi-Fi or cellular coverage for those without in-home Wi-Fi. The iPad was paired with the Fitbit activity monitor and signed into a study-specific Fitbit account that was anonymized to improve confidentiality. The iPad was also signed into a study-specific Zoom account as well as the participant's Companion app account.

**Figure 2:**
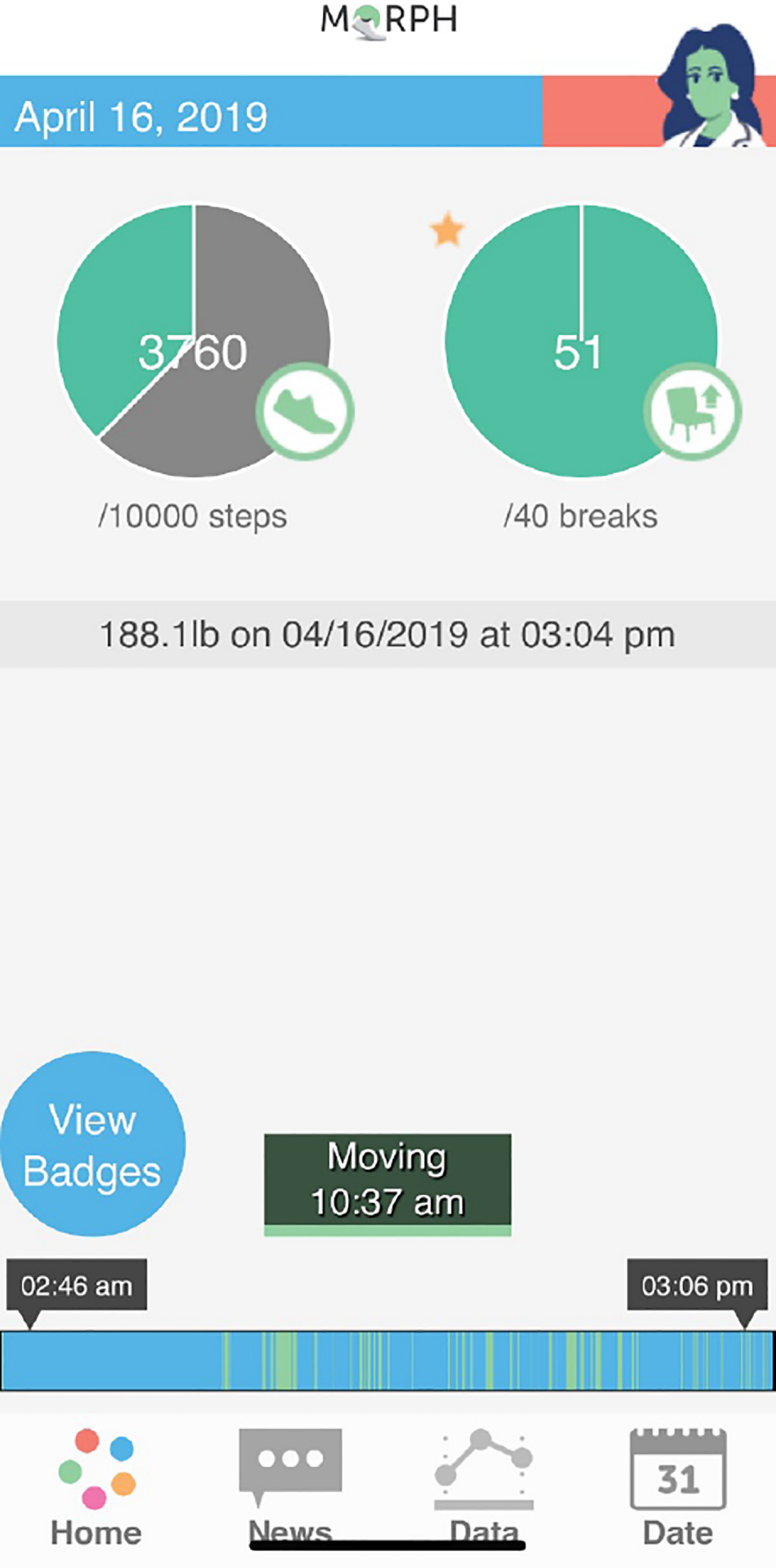
Example Companion App screen.

The MORPH Companion app is a set of mHealth tools that has been refined across several randomized trials (21, 27, 34) that each leveraged user-centered design practices [see references (21, 27, 34]). These tools are designed to support socially rich group-mediated interventions by (1) providing ongoing connection to peers and group-leaders between meetings *via* asynchronous chat; (2) providing unique visual feedback based on objective real-time data to support movement throughout the day; and (3) calling attention to behavioral successes as they occur *via* “mastery badges” (34) to support self-efficacy *via* mastery experiences. Regarding feedback on patterns of activity across the day, the Companion app integrates Fitbit data in near real time, with a primary feedback mechanism being a daily “timeline bar” that resides on the bottom of the app's splash screen (see blue and green bar near the bottom of Figure 2). Here, periods without Fitbit-detected movement are coded as blue as a marker of inactivity, and minutes with Fitbit-detected steps are coded as green. Participants are encouraged to achieve a “tree rings profile” comprised of frequent stripes of green throughout the full day rather than fewer more sustained periods of green. The Companion app also provides participants with an objective daylong activity goal setting infrastructure *via* three “periodic step goals.” Here, participants receive an overall daily stepping goal, and may earn up to 40% of a “step allowance” before 12:00pm, another 40% between 12:01pm and 5:30pm, and a final 40% after 5:31pm. As such, an individual must achieve a minimum of 20% of their daily steps during any one period to achieve their daily goal. More details on this technology kit can be found in the protocol publication (24). Generally, weekly activity goals were set around an increase of approximately 20% weekly (e.g., an additional 800 steps for someone achieving 4,000 steps per day), and these levels were tailored to individual abilities and progress.

As noted above, previous iterations of the DPA approach produced modest improvements to physical activity volume (21, 27), and we suspected this was attributable to the need for focused coaching on DPA early in the program to foster better understanding of the protocol. Thus, the MORPH intervention in the MORPH-II trial placed a strong emphasis on the value of increased physical activity and decreased sitting. This was accomplished in several ways. First, whereas the first MORPH trial placed greater emphasis on nutrition and weight loss, MORPH-II placed greater emphasis on physical activity content, especially early in the study. Second, MORPH-II employed a student assistant coaching model as a low-cost means of providing one-on-one coaching early in the program. Pre-health students interested in interacting with participants first completed a half-semester intensive training bootcamp designed to provide an in-depth education on key theories of behavior change with an emphasis on dual process (35), social cognitive (25), and self-determinative theories (18, 26). Students designed and piloted their own behavioral sessions and practiced coaching their peers. Those who succeeded in this program were offered the opportunity to shadow graduates of the program and professional behavioral coaches in ongoing physical activity and weight loss trials for older adults. Finally, students were selected by these coaches to continue to assistant coaching roles. These student coaches were under the direct oversight of the professional behavioral coach, initially communicating with the rest of the coaching team *via* project management software, and later *via* weekly video conference meetings (a change made in response to participant feedback as detailed below). At a minimum, coaches were expected to see a minimum of one wave of participants through the study and were assigned 1–3 individuals per wave to coach. During each call, coaches followed a rubric of key items to review, including (1) a day-by-day review of previous physical activity feedback within the Companion app, (2) goal review and revision, and (3) troubleshoot of barriers, such as feelings of boredom or aversion to the participant's current physical activities. This information was tracked and reviewed during weekly coaching team meetings.

During MORPH-II, these coaches completed brief individualized coaching calls designed to reinforce participants' daylong movement goals. These were completed in a tapered fashion such that coaches attempted to complete 3 brief weekly calls in weeks 1–3, 2 brief weekly calls in weeks 4–6, and 1 weekly call in weeks 7–12. During each call, coaches asked participants to view their timeline bar from the previous several days. These were reviewed in-depth, identifying periods where the participant moved often, and working to identify consistent daily periods without movement. The pair then collaborated to generate methods of moving during these sedentary periods. During the first call of each week, the pair reviewed the previous week's activity goals and set a goal for the coming week. These calls also offered an opportunity for coaches to address any technological challenges or other concerns related to the program. The coaches also would join the Zoom meetings at the start of each wave to ensure participants had no difficultly joining the meeting, reducing technical disruptions during group sessions.

## Outcomes

Primary assessments were collected by research staff at baseline prior to randomization and after the intervention period (12 weeks), with the exception of the ActivPAL, which was worn during the final week of the intervention period. Participants received the ActivPAL *via* mail and received postage-paid return shipping materials. Questionnaires were collected *via* phone by trained study staff members. An important limitation to note is that due to staffing constraints, assessment staff were not blind to randomization, though they did not participate in intervention delivery.

### Feasibility and acceptability

The primary outcomes of *MORPH-II* were feasibility and acceptability. Feasibility was indexed as recruitment yield, attendance at weekly meetings, and retention for follow-up testing. Acceptability of the technological aspects of the MORPH intervention was assessed *via* the System Usability Scale (36). Here, participants respond to 10 questions related to usability of the Companion app toolset (e.g., “I think that I would like to use this system frequently”, or “I found the system unnecessarily complex”). Final scores fall on a 100-point scale, which are interpreted based on percentile scores. Herein we employ the adjective scale developed by Bangor (37) and refined using 30 years of data by Sauro (38). Scores of ≥84.1 indicate “best imaginable” usability, 80.8–84 indicate “excellent” usability, 71.1–80.7 indicate “good” usability, 51.7–71.0 indicate “ok” usability, and scores below 51.7 indicate “poor” usability. Acceptability was also assessed *via* semi-structured qualitative interview upon completion of the 12-week timepoint. These interviews were conducted by an external professional qualitative interview group [the Qualitative and Patient Reported Outcomes (Q-PRO) resource within the Atrium Health Wake Forest Baptist Comprehensive Cancer Center], who approached all MORPH group participants to complete the interviews. Interviews were completed *via* telephone by trained qualitative researchers over an average duration of 44 min. Audio transcripts from the telephone interviews were transcribed verbatim. The interview guide, which was developed collaboratively by the research team and Q-PRO to gauge participant perceptions related to key aspects of the MORPH intervention, can be found in the Supplement. Herein we focus on topics relevant to the conduct of the MORPH study and the use of the Companion App.

### Physical activity and sedentary behavior

Participants wore an ActivPAL 4 (PAL Technologies Ltd, Glasgow, UK) thigh-mounted triaxial accelerometer for one week at baseline and during the final week of the intervention period. Relative to the ActiGraph, the ActivPAL provides an excellent assessment of both stepping and sedentary behaviors (39–41). Data were scored using PALBatch software version 8, requiring 20 h of valid wear time to constitute a valid day, and participants with at least 2 days of data were retained in analyses. Focal outcomes included average daily steps across valid days, average daily postural transitions (i.e., a count of transitions from sedentary to non-sedentary postures), and average daily total sedentary time. Additionally, because the negative health effects of sedentary time appear related to the presence of sustained bouts of sedentary time rather than overall sedentary time, we also investigated average daily minutes spent in bouts of sedentary time less than 60 min in duration and in bouts greater than 60 min in duration.

### Pain

Pain intensity was assessed using the Patient Reported Outcomes Measurement Information System (PROMIS) (42) 3-item pain intensity scale (version 2). Pain interference was assessed using the PROMIS 8-item pain interference scale. Scores from each scale were uploaded to the PROMIS scoring system, which produces standardized scores wherein 50 represents the national average, with 10 points representing a standard deviation. On each of these scales, higher scores represent greater pain intensity or interference.

### Psychosocial mechanisms of behavior change

As noted above, the MORPH program is designed to enhance autonomy, competency, and relatedness, as these are key drivers of behavior uptake and well-being (17). Participants completed the brief psychological need satisfaction and need frustration scale (BPNSNF), comprising 24 items that are completed on a 1–5 scale where 1 indicates “not at all true” and 5 indicates “completely true”. The scale provides 6 subscales—the three core needs of autonomy, relatedness, and competence, with satisfaction and frustration scores for each—captured as the sum of 4 items per subscale. An example item from the autonomy satisfaction subscale is “I feel a sense of choice and freedom in the things I undertake.” An example item from the relatedness frustration subscale is “I feel that people who are important to me are cold and distant towards me.” As such, higher satisfaction subscale scores indicate greater satisfaction, and higher frustration subscale scores indicate greater frustration.

## Analyses

First, we present a narrative summary of key protocol changes implemented between waves. To characterize the sample and measures of feasibility and system usability, we present simple descriptive statistics, including mean and standard deviation for continuous variables, and a count and percentage for categorical variables. For exploratory analyses of physical activity, sedentary behavior, pain, and psychosocial mediators of behavior change, we conducted a series of analyses of covariance (ANCOVAs). Each model included group assignment as the independent factor, the outcome of interest at week 12 as the dependent variable, and the baseline value for this outcome as a covariate. For each model, we confirmed normality of residuals and homogeneity of variances. In all analyses we focus on effect sizes, given the exploratory nature of these pilot analyses and the small size of the sample, following Cohen's guidelines (43) such that for each ANCOVA, an *η*^2^ = 0.01 was interpreted as a small effect, *η*^2^ = 0.06 as a medium effect, and *η*^2^ = 0.14 as a large effect.

Regarding qualitative analyses, two members of the qualitative research team reviewed transcripts and collaboratively developed a codebook to capture concepts found in the textual data. Codes focused on components related to conduct of the MORPH program, but also captured related concepts discussed by participants such as motivation, connection, and comfort with technology. These data were managed with ATLAS.ti software. Two members of the research team independently coded one-third of the transcripts and then met to compare their schemas. The codebook was adjusted as needed, based on discussions of code meanings and application. Once no further adjustments were needed, one of the researchers completed the coding while the second reviewed their work, noting agreement or disagreement. Discrepancies were discussed and resolved on a rolling basis. Segments of text were reviewed by code and summarized, and these summaries were synthesized into themes using principles of reflexive thematic analysis (44).

## Results

### Participant characteristics

**Figure 3:**
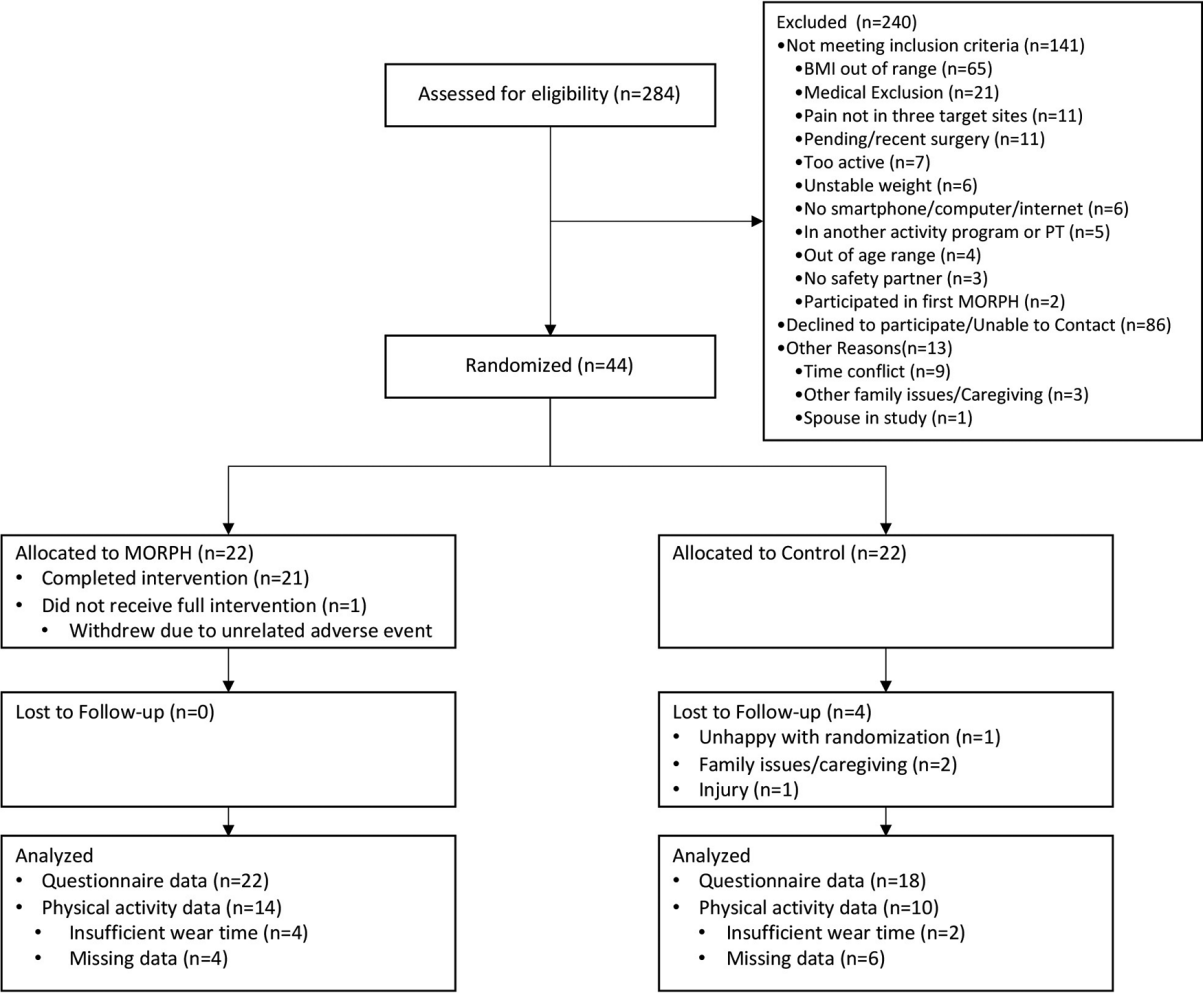
CONSORT diagram.

Participant characteristics as well as baseline pain ratings and activity behaviors are displayed in Table 1. The flow of participants through all study procedures is given in a CONSORT diagram in Figure 3. Briefly, participants (*M* ± SD) were 68.85 ± 7.91 years of age, 75% were female, 75% identified as White and 20.5% identified as Black, and most (90.9%) had a college education. At baseline, pain intensity ratings were approximately one standard deviation above the national average (60.24 ± 4.20), and pain interference ratings were 0.77 standard deviations above national average (57.70 ± 5.35). Participants achieved 4806 ± 2,421 steps per day on average, engaged in 45 ± 16 sit-to-stand transitions, and were sedentary for 10.8 h per day (648.53 ± 188.85 min). A total of 17 intervention participants completed follow-up interviews (see Table 2 for demographic information). These participants were 70.42 ± 6.91 years of age on average, 70.6% were female, 76.5% identified as White and 17.6% identified as Black, and most had a college education (94.1%).

**Table 1:** Participant characteristics, pain, activity behaviors, self-determination theory measures at baseline. *M*, mean, SD, standard deviation.

	MORPH *n* =** **22	Control *n* = 22	Overall *N* = 44
Age; *M* (SD)	68.55 (8.02)	69.14 (7.98)	68.84 (7.91)
Sex; *N* (%)			
Male	5 (22.7)	6 (27.3)	11 (25)
Female	17 (77.3)	16 (72.7)	33 (75)
Race; *N* (%)			
White	17 (77.3)	16 (72.7)	33 (75)
Black	3 (13.6)	6 (27.3)	9 (20.5)
Other	2 (9.1)	0 (0)	2 (4.5)
Lives Alone; *N* (%)	7 (31.8)	8 (36.4)	15 (34)
Married; *N* (%)	9 (40.9)	12 (54.5)	21 (47.7)
College Education; *N* (%)	21 (95.5)	19 (86.3)	40 (90.9)
Pain Intensity; *M* (SD)	60.26 (4.77)	60.22 (3.64)	60.24 (4.20)
Pain Interference; *M* (SD)	58.76 (4.72)	56.63 (5.82)	57.70 (5.35)
Steps; *M* (SD)	4935.14 (2731.83)	4670.40 (2107.46)	4806.00 (2420.55)
Transitions; *M* (SD)	42.24 (17.11)	47.30 (15.03)	44.71 (16.13)
Total Sedentary Time; *M* (SD)	623.63 (244.61)	674.67 (103.43)	648.53 (188.85)
Brief Sedentary Time, *M* (SD)	477.85 (111.15)	391.91 (129.21)	442.04 (124.05)
Extended Sedentary Time, *M* (SD)	184.00 (107.38)	275.02 (135.67)	221.92 (125.78)
Autonomy Satisfaction, *M* (SD)	16.73 (2.45)	15.95 (3.44)	16.34 (2.98)
Autonomy Frustration, *M* (SD)	9.00 (3.80)	9.41 (4.16)	9.20 (3.94)
Relatedness Satisfaction, *M* (SD)	17.91 (3.24)	18.09 (2.58)	18.00 (2.89)
Relatedness Frustration, *M* (SD)	5.55 (2.34)	5.59 (2.32)	5.57 (2.31)
Competence Satisfaction, *M* (SD)	16.95 (1.70)	16.95 (3.09)	16.95 (2.47)
Competence Frustration, *M* (SD)	7.05 (2.28)	7.27 (3.38)	7.16 (2.85)

**Table 2:** Demographics for those who completed the qualitative interview.

	Overall *N* = 17
Age; *M* (SD)	70.42 (6.91)
Sex; *N* (%)	
Male	5 (29.4)
Female	12 (70.6)
Race; *N* (%)	
White	13 (76.5)
Black	3 (17.6)
Other	1 (5.9)
Lives alone; *N* (%)	4 (23.5)
Married; *N* (%)	8 (47.1)
College education; *N* (%)	16 (94.1)

### Key protocol modifications

Three key protocol modifications were made during the conduct of MORPH-II. First, it became apparent in the initial waves of the study that participants would benefit from a second debrief on the technology kit during the first meeting of the program, and this was added to wave 4 of MORPH-II. This was especially beneficial for participants who may have received the technology orientation many weeks prior to the start of the intervention. Second, as an added measure of confidentiality, the participant names that were displayed during video conference meetings were initially anonymized (e.g., “MORPH 6”). Participants in the first two waves of the study opposed this, sometimes strongly. For instance, one noted “You need to have a name. A first name is just fine…it's dehumanizing. We’re not guinea pigs.” Beginning in wave 3, participants were able to display their own name within the software. Finally, preliminary participant feedback indicated heterogeneity in student coaching fidelity such that several student coaches were not routinely reviewing key pieces of physical activity feedback (e.g., the Companion timeline bar), resulting in poor understanding of the DPA goals. In response to this feedback and prior to the start of wave 4, student coaches received retraining, and weekly student coaching meetings shifted from asynchronous to a synchronous format, with greater oversight from senior student coaches and the professional behavioral coach.

### Feasibility

Participants in the MORPH intervention condition attended 82.5% of sessions on average. There was one individual in the intervention condition who dropped during the intervention period in response to a medical event, and session attendance was 85.3% without this individual. In total, $20,114.01 was spent on advertising *via* targeted postcard mailings ($13,894.01) and digital and print newspaper advertisements ($6,220.00). Postcard advertisements yielded 69 contacts, for a cost per contact of $201.36. Newspaper advertisements yielded 34 contacts for a cost of $182.94 per contact. Top-yielding free sources of recruitment included a research-oriented newsletter sent to older adults who had consented to being on the mailing list (47 contacts), and referral from either friends/family or from research coordinators within the local health system (67 contacts). In total, members of the research team attempted to telephone screen 284 individuals and 44 participants (15.5%) converted to randomization. The most common reasons for individuals not being randomized include an inability to contact the individual or a lack of interest (*n* = 86), a BMI that is out of range (*n* = 65), or other medical exclusion (*n* = 21). Of those who were randomized, 18 control participants returned for week 12 testing, and all 22 intervention participants completed follow-up testing, for an overall retention rate of 90.9%.

### Acceptability

**Figure 4:**
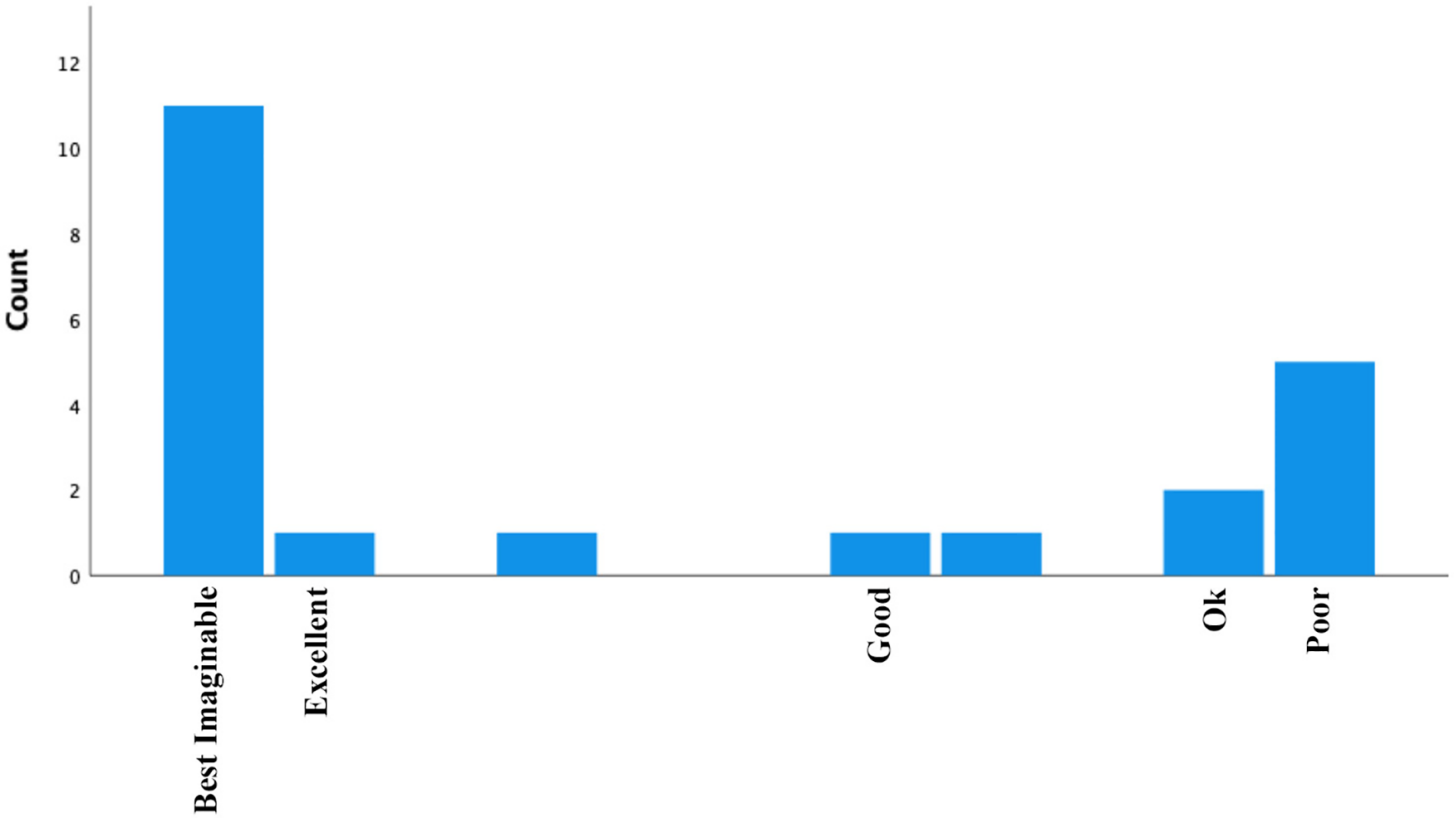
A histogram of system usability scale scores.

A histogram depicting system usability scale scores is provided in Figure 4. The average score was 77, which is classified as “good-to-excellent”, and the median score was 85, which is categorized as “best imaginable”. In total, 11 participants provided scores within the “best imaginable” range, 1 within “excellent”, 3 within “good”, 2 within “ok”, and 5 within “poor”. An exploratory independent-samples t-test comparing those with “ok” or “poor” scores against those with “good” or better scores indicated no statistically significant difference due to age (*p *= 0.21). It is notable that except for one participant in wave 5, the remaining 7 participants with “okay” or “poor” scores were in waves 1–3 of the study prior to student coach retraining.

#### Qualitative feedback related to the overall program experience

Key themes, findings, and takeaways from qualitative interviews are summarized in Table 3. Post-program qualitative feedback revealed the majority of participants gave positive feedback on the MORPH-II program, describing it as “beneficial” and “life-changing”, noting it met or exceeded their expectations. For instance, one participant noted: “*It's a life-changing program. If you want to get well, get off of all of your pain medication and your anti-anxiety medications, this is the program to work with.*” Participants specifically learned that movement can help with pain management, and that it does not need to comprise a formal “workout”: “*[I]t didn’t occur to me until we started doing all this stuff that I was just not moving. The reason I was hurting so bad was because I wasn't moving. It just was a simple matter of getting out of the chair.”* Notably, several participants expressed a desire for more nutrition and weight loss content.

**Table 3:** Key themes, findings, and takeaways based on participant interviews.

Theme	Finding	Take-Away
Awareness	Understanding the pain and movement relationship	Technology-supported programs focused on daylong physical activity can help older adults with chronic pain to understand person physical activity patterns and how these relate to the experience of pain.
Technology	Loss of credit for activity	Intervention tools that fail to acknowledge beneficial behaviors should be avoided. For instance, feedback can acknowledge a participant's total daily step alongside morning, midday, and evening steps.
	Leveraging existing technologies	To minimize participant burden, technological tools should be made available on multiple common platforms (e.g., iOS and Android) when possible.
	Portability	Tablet computers provided good usability for videoconferencing and periodic viewing of feedback. Feedback designed to be viewed often—such as patterns of daily movement—should be available in a more portable format (e.g., smartphone, smart watch)
Bond formation	Value of social connection	Participants valued their ability to develop social connections with group members, the coach, and student coaches.
	Extended duration of bond formation	Participants should be educated to expect that bonds form more slowly over videoconference to normalize this experience.
		To encourage bond formation, remote meetings should open in small-group breakout activities, and extended lecture should be avoided.
Coaching	Value for building awareness	Coaching from pre-health students can be a more cost-efficient way to help participants better understand and adhere to a daylong physical activity goal.
	Importance of close oversight	Implementation of a student coaching model requires close oversight and peer support to ensure coaching fidelity.

#### Qualitative feedback related to the companion app

Regarding feedback related to the Companion app, all but one participant, who reported not using the app often, felt the app helped them to better understand their physical activity behaviors. Participants generally saw value in the visual representation of movement patterns, and in being able distinguish between morning, midday, and evening steps. For instance, one participant noted: “*I liked the little bar at the bottom that showed—the green and blue bar that showed when the activity was more concentrated during the day, and when it wasn't…[I]t helped remind me to get up and move more and not sit still for long periods of time.”* Another noted: “*With the blue line on the bottom and it was very helpful to look back and see, ‘Oh, my goodness. I sat really a long time.’ Oh, yeah, so that was very helpful.*” Several participants directly noted the motivation and encouragement arising from the mastery badges. For instance, one noted “*I loved, loved, loved the rewards. That's encouraging and motivating…I love the Dirty Shoes Award*”, in reference to the badge earned for achieving the daily activity goal.

This report also revealed several areas to be addressed in future uses of the Companion app. For instance, several participants from the initial three waves of the study expressed limited understanding of the timeline bar and periodic step goals. Because the intervention in these waves was delivered prior to student coach retraining—a step that, among other improvements, emphasized reviewing participants' comprehension of these features—it underscores the importance for ongoing evaluation of comprehension and training in use of key mHealth tools in technology-supported interventions. Additionally, while participants generally appreciated the feedback related to reaching movement goals during the morning, midday, and evening, several felt frustrated to lose “credit” for taking extra steps during any given period of the day. For instance, one said: “*I was a little frustrated that if you got over that amount of steps in the morning, that those didn’t count. Because there were times when I lost, it felt like to me, I lost those three hundred or so steps because it was too many for that morning, but I do understand the point of that*”. One potential method for addressing this frustration is to display participants’ total step counts during each period to satisfy the desire to have all steps represented, while separately displaying periodic step goal feedback. Two participants noted that learning the technological aspects of the study was a challenge. One noted: “*At my age, I don’t do a lot of stuff. I don’t have an email address. I don’t text. I’m just old fashioned. That was probably the biggest challenge for me*.” Two participants noted they were Android users and felt it difficult to move to the Apple ecosystem. While we provided iPads to all users to ensure a consistent and secure experience, the Companion app is a native web app in structure and as such is device agnostic. Future implementations may consider providing multiple options to suit preferences or may allow participants to utilize a personal device. Finally, several participants noted that it would be valuable to have tools to view the timeline feedback using a device that is more portable than the tablet. For instance, one stated: “*If that MORPH app had a bit on my wrist it would've been wonderful. That would've really been successful for me ‘cause I could've looked down and said, ‘Oh look, you only got—it's too blue. Get up there and start moving around and making it some more green or whatever, yeah green.’”*.

Participants generally found the Fitbit useful, especially as it provided data to the MORPH app, and did not find the data provided by the Fitbit alone to be as valuable. For instance, one noted: “*What was helpful on the MORPH app that I didn’t have on the Fitbit was the number of steps for each period—morning, afternoon, and evening. That was helpful. It was just a bit richer component of information than what I was just getting from the Fitbit.”* Several participants expressed frustration that they felt the Fitbit poorly captured non-ambulatory movement, such as cycling or doing yardwork, and one did not like the wrist strap that came with the device.

#### Qualitative feedback related to the remote group sessions

Qualitative feedback revealed that most participants had a positive overall experience in the remote group sessions in MORPH, noting it was an important part of the program. One stated “*If you’re in a group study…you eventually develop relationships with those people. You understand what's going on and how they feel and what works and what doesn’t work for them. You learn from that. Also, even though some days you feel horrible, it gives you a sense of camaraderie that you’re sharing with people who totally get what you’re going through. That's nice. Very important.”* Still, several people noted that developing social bonds was more challenging *via* videoconference relative to face-to-face meetings, stating “*I mean it took a lot longer, of course, on a Zoom to—the first couple of weeks, I don’t think we had any connection much with each other. Then, but after that, you sort of get to talking a little bit more, and get to know the people a little bit.”* Others did not feel as strongly that their group connected: “*I’m not sure I would really call them group meetings…I thought after a few weeks, we’d be interactive as a group, and I don’t think we were terribly interactive as a group. I think the purpose of that is to be, but we weren’t very good at it.”* This is an expected outcome from small group meetings, which are affected by group composition. Future implementations of MORPH programming may benefit from the collection of participant feedback early in the program to better match the needs of each group. Participants also expressed desire for more formal structures for transitioning the Zoom sessions to participant control on completion of the 12-week intervention period, feeling unprepared to continue the calls with others in their group. Finally, several participants noted a desire for more options for meeting times, or for somewhat shorter meetings. One noted: “*Our group was small enough that we didn’t need an hour. That was too much, and it's every week.”*

#### Qualitative feedback related to student coaching calls

As described above, feedback from the initial three waves of the study indicated inconsistent student coaching. Approximately half of the participants interviewed indicated their coaches consistently reviewed daily physical activity behaviors, encouraged successes, and helped to address challenges. One stated “*The coach that calls you, that is very specific to you. You’re not in a group. It's just specific to you as an individual, and that was, I think—you cannot take that out of the program.”* Another underscored the value of the positive encouragement their coach provided: “*It gave me an opportunity to say what I felt like I was doing positively and yet, also get some positive support for the efforts I was making. I feel positive about the student's contact…Actual praise and the fact that they asked how I was feeling. I would tell ‘em how I was doing. They verbalized their support for the activity that I was doing and the attempts that I was doing with dietary management.”* Those who received only general check-in calls did not find as much value in them*: “Super nice young ladies and very good listeners. It was not totally clear to—most of us in the group said that, asked what their purpose was to calling us. They just said, ‘Well, how are you doing? Do you have any questions or any problems?’”* Most participants expressed positive feedback on the coaches as personable and effective listeners, and one stated they bonded more with the student coach than anyone else in the study. Two participants noted it was somewhat difficult to bond with their students due to age differences. Other feedback related to logistics included a desire for student coaches to participate actively in the group sessions, and to dedicate an initial coaching call to developing a call schedule with the participant. These changes were made following the third wave of the study.

### Intervention effects on pain, physical activity, and psychosocial outcomes

**Figure 5:**
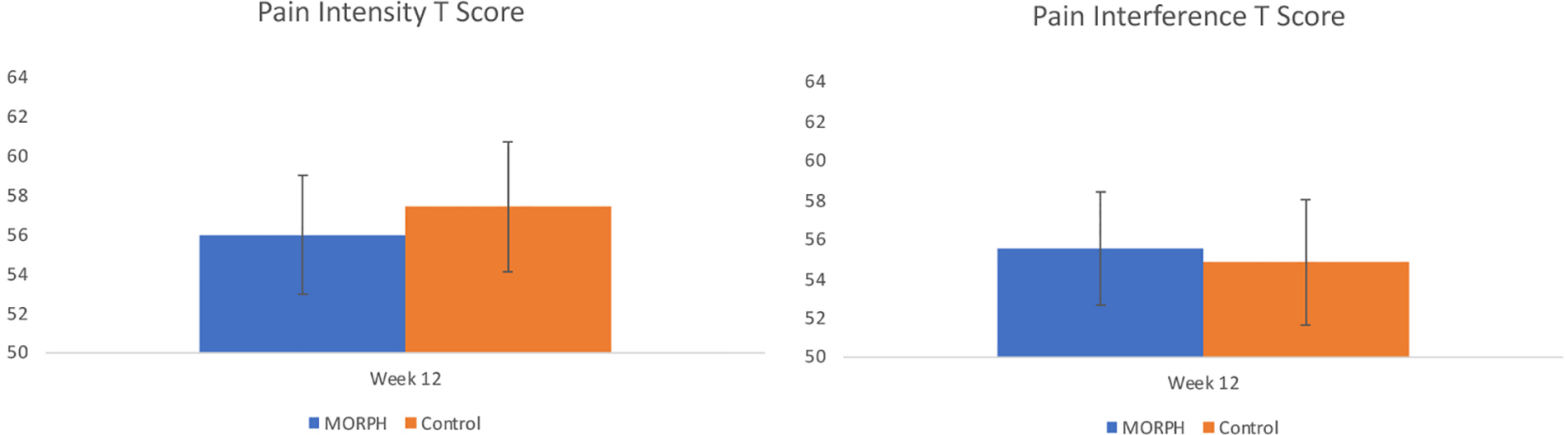
Baseline-adjusted pain intensity and pain interference *T* scores at week 12.

**Figure 6:**
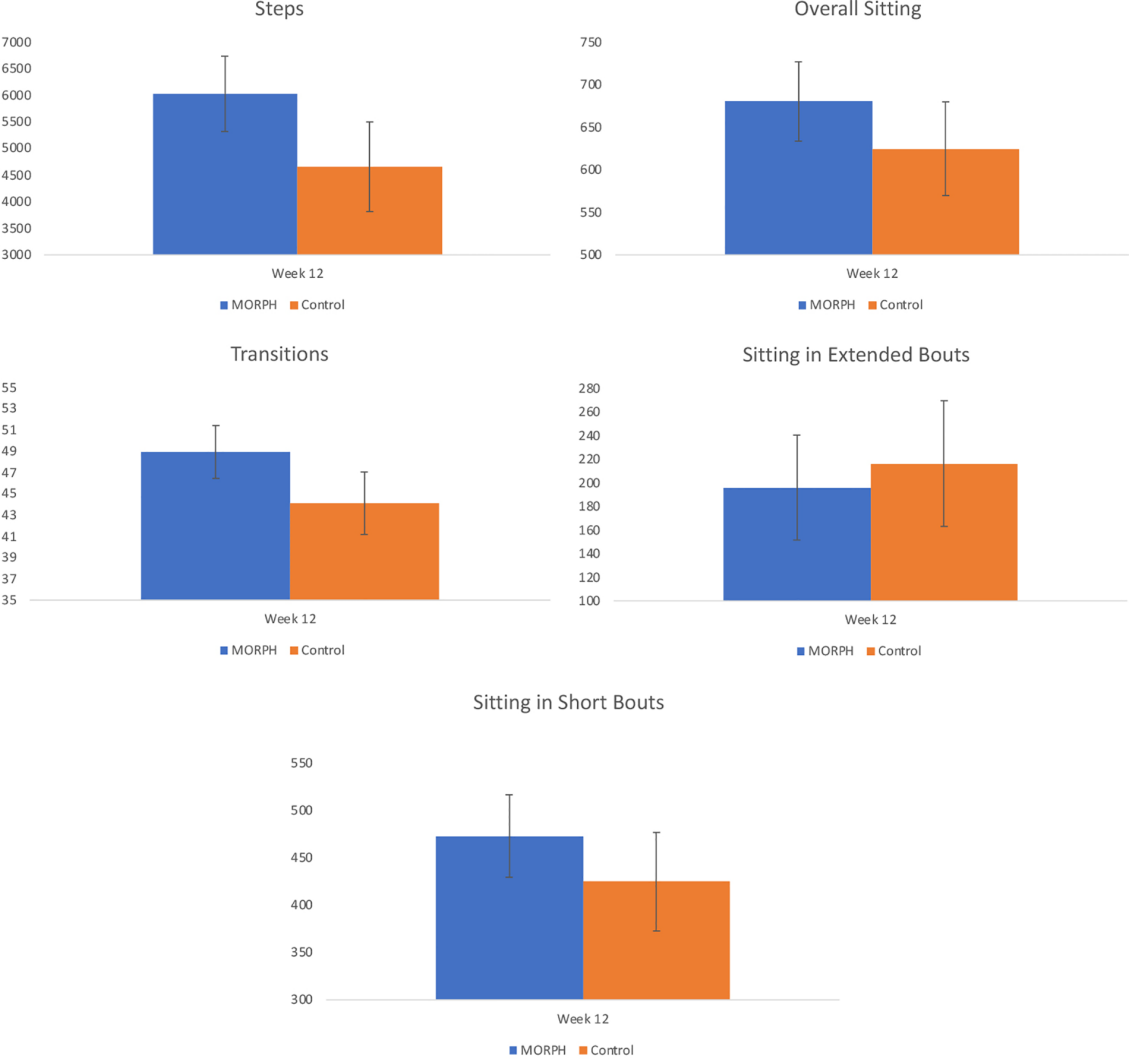
Baseline-adjusted markers of physical activity and sedentary behavior collected *via* ActivPAL at week 12.

**Figure 7:**
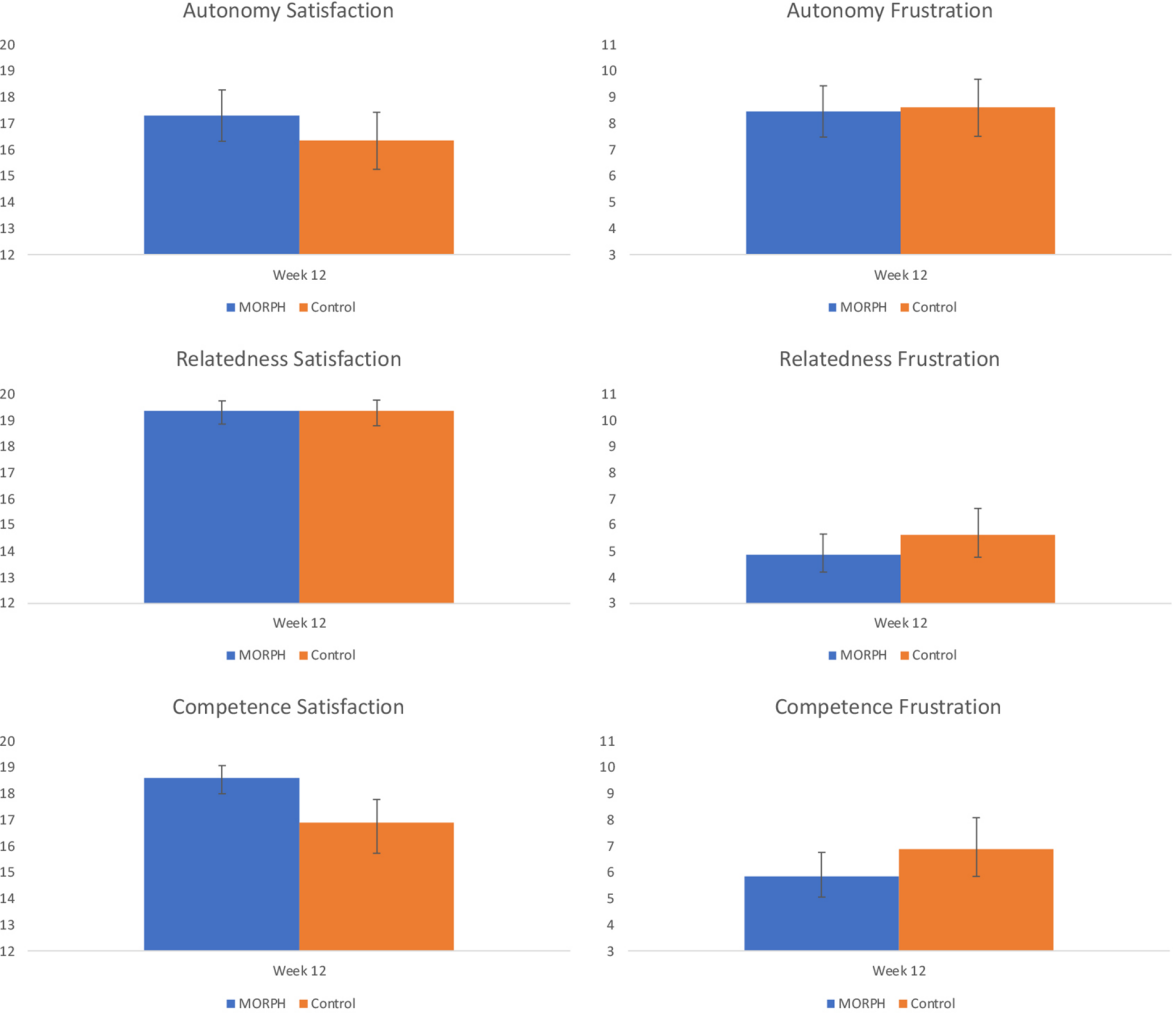
Baseline-adjusted basic psychological needs satisfaction and frustration scores at week 12. Note, relatedness and competence scores were natural log transformed (reflected for satisfaction scores) to account for skewed residuals. Back-transformed values are presented here.

Baseline-adjusted scores, *p* values, and effect sizes from each ANCOVA model are displayed in Table 4. Baseline-adjusted values are displayed in Figures 5–7. Regarding pain intensity, there was a small effect (*η*^2^ = 0.01) favoring the intervention condition. Regarding pain interference, the ANCOVA indicated there was no group effect (*η*^2^ = 0.01). As depicted in Figure 5, both conditions improved pain scores from baseline. We replicated this analysis using raw pain interference scores rather than T-scores, as the ANCOVA revealed a violation of homogeneity of variances (Levene's *p* = 0.02) driven by less variability in week 12 pain interference T scores among intervention participants relative to control. Utilizing the raw scores did not violate this assumption (Levene's *p* = 0.24), and interpretation did not differ.

**Table 4:** Baseline-adjusted scores at week 12; *p* values and effect sizes were obtained from ANCOVA models. *Transformation applied due to heterogeneous variances or non-Normal residuals. Back-transformed data presented here. Short sedentary time captures average daily minutes sedentary bouts of less than 60 min, and extended sedentary time captures average daily minutes in abouts of at least 60 min.

	Adjusted week 12 score	*p*	*η* ^2^
Pain Intensity		0.52	0.01
MORPH	56.00 (53.00,59.00)		
Control	57.42 (54.10, 60.74)		
Pain Interference		0.76	<0.01
MORPH	55.50 (52.63,58.38)		
Control	54.83 (51.64,58.02)		
Steps		0.02	0.23
MORPH	6022.84 (5319.75, 6725.93)		
Control	4656.12 (3817.16, 5495.09)		
Transitions		0.02	0.24
MORPH	48.93 (46.44, 51.43)		
Control	44.10 (41.15, 47.05)		
Sedentary Time		0.12	0.11
MORPH	680.26 (633.89, 726.64)		
Control	624.50 (569.63, 679.38)		
Short Sedentary Time		0.17	0.09
MORPH	472.87 (429.33, 516.42)		
Control	425.04 (372.96, 477.12)		
Extended Sedentary Time		0.56	0.02
MORPH	195.66 (151.20, 240.12)		
Control	215.90 (162.66, 269.13)		
Autonomy Satisfaction		0.20	0.05
MORPH	17.31 (16.33, 18.29)		
Control	16.35 (15.26, 17.43)		
Autonomy Frustration		0.85	<0.01
MORPH	8.47 (7.49, 9.44)		
Control	8.60 (7.52, 9.68)		
Relatedness Satisfaction*		0.97	<0.01
MORPH	19.36 (18.87, 19.74)		
Control	19.35 (18.79, 19.77)		
Relatedness Frustration*		0.19	0.05
MORPH	4.86 (4.19, 5.64)		
Control	5.63 (4.78, 6.63)		
Competence Satisfaction*		<0.01	0.22
MORPH	18.58 (17.98, 19.06)		
Control	16.88 (15.74, 17.78)		
Competence Frustration*		0.14	0.06
MORPH	5.84 (5.04, 6.76)		
Control	6.88 (5.85, 8.08)		

Regarding ActivPAL-measured physical activity (see Figure 6), it is notable that 24 individuals had at least two valid days of data at both baseline and w12 (MORPH *n* = 14; see CONSORT for reasons). An ANCOVA revealed a large effect (*η*^2^ = 0.23) for average daily steps favoring the MORPH condition, corresponding to a difference of 1,366.72 baseline-adjusted steps at week 12. Likewise, the ANCOVA model comparing baseline-adjusted postural transitions revealed a large effect (*η*^2^ = 0.24) in favor of MORPH, corresponding to a difference of 4.84 baseline-adjusted daily transitions at week 12. The ANCOVA comparing baseline-adjusted average daily sedentary time revealed a moderate-to-large effect (*η*^2^ = 0.11) favoring the control condition, reflected in a difference of 55.76 min. Further investigation of sedentary bout length revealed this was driven by an increase in time spent in short bouts, reflected in a moderate-to-large effect (*η*^2^ = 0.09) again favoring the control condition, who engaged in 47.83 min less sitting in short bouts daily on average. Regarding long sedentary bouts, the ANCOVA revealed a small effect favoring the MORPH condition (*η*^2^ = 0.02), who, on average, spent 20.24 fewer minutes in sustained sitting bouts compared with control after controlling for baseline.

Our final set of models examined differences in self-determinative needs expected to drive motivated behavior and well-being. Regarding autonomy satisfaction, the ANCOVA revealed a moderate (*η*^2^ = 0.05) effect favoring MORPH. There was no effect present for autonomy frustration (*η*^2^ = 0.01), with both conditions demonstrating reductions in frustration over the course of the intervention (see Figure 7). Both relatedness subscales, as well as both competence subscales demonstrated skewed residuals, and so a natural log transformation was applied to frustration scales and a reflected natural log transformation was applied to satisfaction subscales, which were negatively skewed. Regarding relatedness satisfaction, there was no group effect present (*η*^2^ = 0.01), and both conditions demonstrated small-to-moderate improvements in relatedness satisfaction (see Figure 7). By contrast, there was a moderate effect (*η*^2^ = 0.05) for relatedness frustration favoring MORPH participants. Regarding competence satisfaction, there was a large effect (*η*^2^ = 0.22) whereby MORPH participants demonstrated an improvement in competence satisfaction while control demonstrated a decrease. Finally, there was a moderate-sized effect (*η*^2^ = 0.06) for competence frustration whereby MORPH participants demonstrated less frustration over time and control participants demonstrated a small increase.

## Discussion

MORPH-II iterated upon a DPA intervention and was primarily intended to refine the program for remote delivery and to enhance uptake of the DPA protocol. To this end, a key product of this research is a brief inventory of protocol modifications made iteratively during the study. The modifications were responsive to (1) participants' objection to deidentification as part of the videoconference group; (2) the important role of the individual coaching for developing an understanding of the DPA protocol and the need for synchronous oversight of student coaches providing this service; and (3) the value of participants receiving two opportunities for training on the application interface.

The second key purpose of MORPH-II was to examine its feasibility and acceptability. Support for its feasibility includes high class attendance to the remote sessions (82.5% on average), and the fact that all active intervention participants were retained for follow-up testing. Recruitment costs were fairly high due to two advertising methods (newspaper and targeted postcard mailing) that were expensive with low return. It is also notable that the most common reasons that contacts did not convert to randomization include lack of interest in participating and not meeting BMI criteria. Future iterations of MORPH will explore other targeted and community-based recruitment methods that offer the opportunity to state the scope of the program more clearly. Both quantitative and qualitative data reflect the high degree of acceptability of the MORPH program. Qualitative interviews conducted by an external qualitative research group revealed positive evaluations of MORPH. Most notably, participants commented on a new awareness of how movement and sitting relate to pain, and the value of the unique visual feedback provided within the Companion app for better understanding their daily activity and sitting patterns. Participant feedback supported the value of the group as a tool of behavior change, though participants provided the useful feedback that bond formation happened more slowly *via* videoconference relative to face-to-face interactions. While the study of social bond formation *via* videoconference is in its early days, researchers have put forth several potential causes for slowed bond formation. For instance, individuals may feel stress as they manage conversation without common non-verbal communication cues. Likewise, individuals may experience “hyper-gaze”, or the sensation that one is consistently being watched by all others (45). As such, future work may benefit from including early education related to the longer time course of bond formation to normalize this phenomenon and to help participants navigate any new communication challenges. Additionally, this underscores the importance of encouraging participant interaction early and often by minimizing lecture and by opening each session with small group break-out activities.

Qualitative feedback revealed several other key considerations for future implementations of MORPH. First, as noted above, a key role of the one-on-one student coaching calls within MORPH-II was to provide early and focused review of the feedback provided within the Companion app. We discovered that during the early waves of the study, some student coaches failed to engage in this feedback review, and the qualitative findings revealed that participants enrolled in these early waves were less likely to understand the DPA protocol and associated feedback within the digital health toolset. Second, as MORPH-II was focused on enhancing physical activity levels, several participants noted a desire for more weight loss and nutrition content. And third, participants generally found value in the periodic goal feedback, which subdivided their daily step goal into morning, midday, and evening periods. Still, some expressed that they felt frustrated they did not receive credit for engaging in more activity during a given period, as excess steps did not count toward the goal (i.e., graphical feedback would no longer display steps above 40% of the daily goal during any period). It may be that providing a visual feedback element capturing these “bonus” steps may help to address this frustration, and additional user-centered design is warranted.

As a final marker of acceptability, participants provided system usability scale scores for the Companion app. These scores most often fell into the “best imaginable” category, and average scores were “good-to-excellent”. Importantly, all but one of the poor usability scores were provided by participants enrolled in the first three waves of the study. This provides further indication of the value of the brief individual calls for building an understanding of digital health tools as they relate to DPA goals.

Finally, we examined the preliminary impact of the MORPH-II program on pain, objectively assessed physical activity and sedentary behaviors, and autonomy, competence, and relatedness, in comparison with a control condition who received brief monthly contacts and the Fitbit and scale. Intriguingly, both conditions improved on measures of pain intensity and interference. There may be several potential explanations for the improvements in pain symptoms observed in the control participants. It is notable that questionnaires were collected *via* interview, which may produce greater response bias than self-administered questionnaires (46). Alternatively, these changes may be related to changes in activity and sedentary behaviors over the study period. Indeed, those in the control condition decreased time spent sedentary, though they also decreased daily steps. By contrast, MORPH participants increased their daily steps and demonstrated more daily postural transitions. Experimental evidence suggests that physical activity and sedentary behaviors affect different aspects of pain modulation (12), and so it may be that each group reported improved pain symptoms in response to changes in physical activity or sedentary behaviors.

The beneficial effects of the MORPH-II program on steps and postural transitions are especially promising, given the present focus in the sedentary behavior literature on avoiding sustained sitting—as opposed to reducing daily volume of sitting—to improve health. For instance, a report from the 2018 Queensland Sedentary Behavior Think Tank, comprising leading sedentary behavior researchers, emphasized the importance of avoiding prolonged and static sitting *via* physical activity and postural transitions, noting this may be especially important for older adults (47).

Finally, MORPH-II was designed to enhance one's sense of autonomy by training individuals to select activities to suit their daily preferences, competence through goal progression and the provision of real-time feedback, and relatedness *via* bonding within the group. Our results indicate this design was successful. Relative to the control condition, MORPH participants expressed better autonomy and competency satisfaction, and less relatedness and competency frustration. These initial findings are encouraging and bode well for the impact of a program like MORPH on long-term behavior change as well as quality of life (17).

### Strengths and weaknesses

The MORPH-II pilot trial had several key strengths we would like to emphasize. First, it was a randomized controlled pilot trial that was delivered fully remotely, allowing for participation by those would not otherwise have the ability to commute weekly to an academic medical center. It also leveraged a low-cost student coaching model that allowed for cost-efficient and intensive individualized coaching while simultaneously meeting students' desire to engage with research participants. In addition to continued refinement of this coaching model, a key next step lies in disseminating our coach training and oversight resources so that they may be leveraged by other community and academic research institutions interested in implementing similar programs. MORPH-II provided participants with a technology kit, which included an evidence-based mHealth toolset designed to support self-regulation and social connectivity. This kit was prepared so that participants could use it out of the box, and participants engaged in an orientation meeting designed to foster self-efficacy for engaging with the technology. MORPH-II also employed an iterative refinement structure, which allowed for the identification of protocol limitations, and subsequent piloting of modifications designed to address these limitations. This also allows for the publication of key lessons learned that are actionable by others interested in conducting similar trials. An external qualitative research service conducted all interviews and qualitative analyses. Finally, 25% of the sample was non-White, which is a greater proportion than in the first MORPH pilot (17.9%) and may reflect the ability for remote interventions to reach a more diverse group of older adults.

There are also several key limitations that must be acknowledged. The pilot intervention was brief at only 12 weeks, and as such it is difficult to determine the durability of any findings discussed herein. Participants were required to have an internet-connected device at entry to facilitate baseline testing, which led to the exclusion of six potential participants and potentially limits generalizability. As the focus of the study was on refining the MORPH-II protocol, the sample size was small, and as such any statistical analyses must be interpreted with caution. Likewise, the small sample precluded our ability to investigate whether the impact of MORPH-II varied by key sociodemographic factors such as sex, gender, race, income, or education. Moreover, due to insufficient wear-time, losses during post, and device malfunction (especially battery failure), we only had ActivPAL data on a subset of participants. Finally, while assessors did not participate in the intervention, they were not blinded, which may introduce bias into the pain and self-determinative outcomes. Given the promising findings reported herein and observed in the initial MORPH pilot trial (23), and in recognition of the limitations to each, an important next step in evaluating the MORPH intervention will be to conduct a fully powered single-blind randomized trial integrating the revised DPA program back into the weight loss program tested in the first MORPH trial, recruiting individuals of diverse sociodemographic backgrounds, regardless of their device ownership status to participate in a longer-term intervention.

## Conclusion

Achieving sufficient levels of physical activity while breaking sustained bouts of sitting impact an array of important health outcomes across the lifespan (48), including the experience of pain (12). MORPH-II accomplishes the important goal of refining and examining the feasibility, acceptability, and initial impact of a unique DPA intervention delivered fully remotely. Our findings indicate that the MORPH-II intervention was feasible and acceptable, and may positively impact steps, postural breaks, and several key domains of basic psychological needs detailed in self-determination theory. MORPH-II participants also expressed improved pain intensity and interference, though these effects were also observed in the control condition. Given the focus of MORPH-II on sustainable physical activity promotion in a package that has broad reach, a key next step lies in establishing the efficacy of the MORPH approach in an adequately powered sample over a longer duration. Success in this effort may guide the development of large-scale interventions offering unique lifestyle pain treatment strategies to those living with chronic pain. Finally, while MORPH's group mediated approach did result in improved relatedness, it is clear that future remotely delivered interventions need to consider including activities early in treatment that increase feelings of togetherness, familiarity with one another, and interpersonal trust.

## Data Availability

The raw data supporting the conclusions of this article will be made available by the authors, without undue reservation.

## References

[1] Tsang A Von Korff M Lee S Alonso J Karam E Angermeyer MC Common chronic pain conditions in developed and developing countries: gender and age differences and comorbidity with depression-anxiety disorders. J Pain. (2008) 9(10):883–91. 10.1016/j.jpain.2008.05.005 18602869

[2] Domenichiello AF Ramsden CE. The silent epidemic of chronic pain in older adults. Prog Neuropsychopharmacol Biol Psychiatry. (2019) 93:284–290. 10.1016/j.pnpbp.2019.04.006 31004724 PMC6538291

[3] Office of the Inspector General. Opioids in medicare part D: Concerns about extreme use and questionable prescribing. New York: U.S. Department of Health & Human Services (2017).

[4] NIH HEAL Initiative 2021 Annual Report | NIH HEAL Initiative (2021). National Institutes of Health. Available at: https://heal.nih.gov/about/heal-annual-report

[5] Krebs EE Gravely A Nugent S Jensen AC DeRonne B Goldsmith ES Effect of opioid vs nonopioid medications on pain-related function in patients with chronic back pain or hip or knee osteoarthritis pain: the SPACE randomized clinical trial. JAMA. (2018) 319(9):872–82. 10.1001/jama.2018.0899 29509867 PMC5885909

[6] Campbell G Noghrehchi F Nielsen S Clare P Bruno R Lintzeris N Risk factors for indicators of opioid-related harms amongst people living with chronic non-cancer pain: findings from a 5-year prospective cohort study. EClinicalMedicine. (2020) 28:100592. 10.1016/j.eclinm.2020.100592 33294810 PMC7700907

[7] Stuck AE Walthert JM Nikolaus T Büla CJ Hohmann C Beck JC. Risk factors for functional status decline in community-living elderly people: a systematic literature review. Soc Sci Med. (1999) 48(4):445–69. 10.1016/s0277-9536(98)00370-0 10075171

[8] Gaskin DJ Richard P. The economic costs of pain in the United States. J Pain. (2012) 13(8):715–24. 10.1016/j.jpain.2012.03.009 22607834

[9] Pain Management Effectiveness Research Network | NIH HEAL Initiative [undated]. Available at: https://heal.nih.gov/research/clinical-research/pain-management-research

[10] Park SM Kim HJ Jeong H Kim H Chang BS Lee CK Longer sitting time and low physical activity are closely associated with chronic low back pain in population over 50 years of age: a cross-sectional study using the sixth Korea National Health and Nutrition Examination Survey. Spine J. (2018) 18(11):2051–58. 10.1016/j.spinee.2018.04.003 29678404

[11] Dzakpasu FQS Carver A Brakenridge CJ Cicuttini F Urquhart DM Owen N Musculoskeletal pain and sedentary behaviour in occupational and non-occupational settings: a systematic review with meta-analysis. Int J Behav Nutr Phys Act. (2021) 18(1):159. 10.1186/s12966-021-01191-y 34895248 PMC8666269

[12] Doré I Plante A Peck SS Bedrossian N Sabiston CM. Physical activity and sedentary time: associations with fatigue, pain, and depressive symptoms over 4 years post-treatment among breast cancer survivors. Support Care Cancer. (2022) 30(1):785–92. 10.1007/s00520-021-06469-2 34387728

[13] Naugle KM Ohlman T Naugle KE Riley ZA Keith NR. Physical activity behavior predicts endogenous pain modulation in older adults. Pain. (2017) 158(3):383–90. 10.1097/j.pain.0000000000000769 28187102

[14] Bass C. The role of emotion in determining pain. Dig Dis. (2009) 27(Suppl 1):16–23. 10.1159/000268117 20203493

[15] Wolf LD Davis MC. Loneliness, daily pain, and perceptions of interpersonal events in adults with fibromyalgia. Health Psychol. (2014) 33(9):929–37. 10.1037/hea0000059 25180546 PMC4214136

[16] Loeffler A Steptoe A. Bidirectional longitudinal associations between loneliness and pain, and the role of inflammation. Pain. (2021) 162(3):930–7. 10.1097/j.pain.0000000000002082 32960533 PMC7886943

[17] Teixeira PJ Carraça EV Markland D Silva MN Ryan RM. Exercise, physical activity, and self-determination theory: a systematic review. Int J Behav Nutr Phys Act. (2012) 9:78. 10.1186/1479-5868-9-78 22726453 PMC3441783

[18] Rejeski WJ Fanning J. Models and theories of health behavior and clinical interventions in aging: a contemporary, integrative approach. Clin Interv Aging. (2019) 14:1007–19. 10.2147/CIA.S206974 31213787 PMC6549388

[19] U.S. Department of Health and Human Services. 2018 Physical activity guidelines advisory committee scientific report (2018). 10.3109/15360288.2015.1037530 26095483

[20] Okifuji A Hare BD. The association between chronic pain and obesity. J Pain Res. (2015) 8:399–408. 10.2147/JPR.S55598 26203274 PMC4508090

[21] Fanning J Rejeski WJ Leng I Barnett C Lovato JF Lyles MF Intervening on exercise and daylong movement for weight loss maintenance in older adults: a randomized, clinical trial. Obesity. (2022) 30(1):85–95. 10.1002/oby.23318 34932885 PMC8711609

[22] Leeuw M Goossens ME Linton SJ Crombez G Boersma K Vlaeyen JW. The fear-avoidance model of musculoskeletal pain: current state of scientific evidence. J Behav Med. (2007) 30(1):77–94. 10.1007/s10865-006-9085-0 17180640

[23] Boutevillain L Dupeyron A Rouch C Richard E Coudeyre E. Facilitators and barriers to physical activity in people with chronic low back pain: a qualitative study. PLoS One. (2017) 12(7):e0179826. 10.1371/journal.pone.0179826 28742820 PMC5526504

[24] Fanning J Brooks AK Hsieh KL Kershner K Furlipa J Nicklas BJ Building on lessons learned in a mobile intervention to reduce pain and improve health (MORPH): protocol for the MORPH-II trial. MIR Res Protoc. (2021) 10(7):e29013. 10.2196/29013 PMC8329761 34279241

[25] Bandura A. Self-efﬁcacy: The exercise of control. New York, NY: W. H. Freeman and Company (1997).

[26] Deci EL Ryan RM. Self-determination theory: a macrotheory of human motivation, development, and health. Can Psychol. (2008) 49(3):182–5. 10.1037/a0012801

[27] Fanning J Brooks AK Ip E Nicklas BJ Rejeski WJ Nesbit B A mobile health behavior intervention to reduce pain and improve health in older adults with obesity and chronic pain: the MORPH pilot trial. Front Digit Health. (2020) 2:598456. 10.3389/fdgth.2020.598456 33817686 PMC8018691

[28] Fanning J Brooks AK Hsieh KL Kershner K Furlipa J Nicklas BJ The effects of a pain management-focused mobile health behavior intervention on older adults’ self-efficacy, satisfaction with functioning, and quality of life: a randomized pilot trial. Int J Behav Med. (2021) 29(2):240–6. 10.1007/s12529-021-10003-3 34018138 PMC8136759

[29] Collins LM Murphy SA Strecher V. The multiphase optimization strategy (MOST) and the sequential multiple assignment randomized trial (SMART): new methods for more potent eHealth interventions. Am J Prev Med. (2007) 32(5 Suppl):S112–8. 10.1016/j.amepre.2007.01.022 17466815 PMC2062525

[30] Collins LM Baker TB Mermelstein RJ Piper ME Jorenby DE Smith SS The multiphase optimization strategy for engineering effective tobacco use interventions. Ann Behav Med. (2011) 41(2):208–26. 10.1007/s12160-010-9253-x 21132416 PMC3053423

[31] Shields M Connor Gorber S Janssen I Tremblay MS. Bias in self-reported estimates of obesity in Canadian health surveys: an update on correction equations for adults. Health Rep. (2011) 22(3):35–45. PMID: 22106788. 22106788

[32] Guralnik JM Simonsick EM Ferrucci L Glynn RJ Berkman LF Blazer DG A short physical performance battery assessing lower extremity function: association with self-reported disability and prediction of mortality and nursing home admission. J Gerontol. (1994) 49(2):M85–94. 10.1093/geronj/49.2.m85 8126356

[33] de Jager CA Budge MM Clarke R. Utility of TICS-M for the assessment of cognitive function in older adults. Int J Geriatr Psychiatry. (2003) 18(4):318–24. 10.1002/gps.830 12673608

[34] Fanning J Roberts S Hillman CH Mullen SP Ritterband L McAuley E. A smartphone "app"-delivered randomized factorial trial targeting physical activity in adults. J Behav Med. (2017) 40(5):712–29. 10.1007/s10865-017-9838-y 28255750

[35] Brand R Ekkekakis P. Affective–Reflective Theory of physical inactivity and exercise. Ger J Exerc Sport Res. (2017) 48:48–58. 10.1007/s12662-017-0477-9

[36] Brooke J. “SUS—a ‘quick and dirty’ usability scale”. In: Jordan PW Thomas B McClelland IL Weerdmeester B, editors. Usability Evaluation In Industry. CRC Press (1996). p. 189–94.

[37] Bangor A Kortum PT Miller JT. An empirical evaluation of the system usability scale. Int J Hum Comput Interact. (2008) 24:574–94. 10.1080/10447310802205776

[38] Sauro J. 5 Ways to Interpret a SUS Score (2018). MeasuringU. Available at: https://measuringu.com/interpret-sus-score/

[39] Kim Y Barry VW Kang M. Validation of the ActiGraph GT3X and activPAL accelerometers for the assessment of sedentary behavior. Meas Phys Educ Exerc Sci. (2015) 19:125–37. 10.1080/1091367X.2015.1054390

[40] An HS Kim Y Lee JM. Accuracy of inclinometer functions of the activPAL and ActiGraph GT3X+: a focus on physical activity. Gait Posture. (2017) 51:174–80. 10.1016/j.gaitpost.2016.10.014 27780084 PMC6331039

[41] Hergenroeder AL Barone Gibbs B Kotlarczyk MP Kowalsky RJ Perera S Brach JS. Accuracy of objective physical activity monitors in measuring steps in older adults. Gerontol Geriatr Med. (2018) 4:2333721418781126. 10.1177/2333721418781126 29977979 PMC6024488

[42] Health Measures. PROMIS [undated]. Available at: http://www.healthmeasures.net/explore-measurement-systems/promis

[43] Cohen J. Statistical power analysis for the behavioral sciences. New York, NY: Academic Press (1988). p 567.

[44] Braun V Clarke V. Using thematic analysis in psychology. Qual Res Psychol. (2006) 3(2):77–101. 10.1191/1478088706qp063oa

[45] Gutman J Gordon I Vilchinsky N. is social presence indeed present in remote social interactions? A call for incorporating physiological measures of synchrony when assessing the social nature of interpersonal interactions via videoconferencing platforms. Front Psychol. (2022) 13:822535. 10.3389/fpsyg.2022.822535 35391982 PMC8980853

[46] Brenner PS DeLamater J. Lies, Damned Lies, and Survey Self-Reports? Identity as a Cause of Measurement Bias. Soc Psychol Q. (2016) 79(4):333–54. 10.1177/0190272516628298 29038609 PMC5639921

[47] Biddle SJH Bennie JA De Cocker K Dunstan D Gardiner PA Healy GN Controversies in the science of sedentary behaviour and health: insights, perspectives and future directions from the 2018 Queensland sedentary behaviour think tank. Int J Environ Res Public Health. (2019) 16(23):4762. 10.3390/ijerph16234762 31783708 PMC6926563

[48] Piercy KL Troiano RP Ballard RM Carlson SA Fulton JE Galuska DA The physical activity guidelines for Americans. JAMA. (2018) 320:2020–8. 10.1001/jama.2018.14854 30418471 PMC9582631

