# Erratum: A remote group-mediated daylong physical activity intervention for older adults with chronic pain: Results of the MORPH-II randomized pilot trial

**DOI:** 10.3389/fdgth.2023.1298782

**Published:** 2023-10-09

**Authors:** Frontiers Production Office

**Affiliations:** Frontiers Media SA, Lausanne, Switzerland

**Keywords:** physical activity, sedentary behavior, pain, aging, technology

An erratum on A remote group-mediated daylong physical activity intervention for older adults with chronic pain: Results of the MORPH-II randomized pilot trial By Fanning J, Brooks AK, Ford S, Robison JT, Irby MB and Rejeski WJ. (2022). Front. Digit. Health 4:1040867. doi: 10.3389/fdgth.2022.1040867

Due to a production error, the reference list in the published paper was missing some citations and did not reflect the authors' corrected manuscript.

The publisher apologizes for this mistake.

The original version of this article has been updated.

