# Supplementary material for: A remote group-mediated daylong physical activity intervention for older adults with chronic pain: Results of the MORPH-II randomized pilot trial
Source: Front Digit Health. 2022 Nov 2;4:1040867. doi: 10.3389/fdgth.2022.1040867 (PMC9666366; doi:10.3389/fdgth.2022.1040867)
Supplement: Supplementary file 1 [file Datasheet1.docx]

Supplementary Material

# Interview Guide

**Interview Guide**

Note to interviewer: This guide is structured into five sections (introduction, recruitment, MORPH components, other study elements, and wrap-up). Within each section are key discussion points, enumerated, followed by additional prompts (bullets; optional) that can be used to foster conversation.

**PID: _______________ Date: _______________ Start Time: ________________**

**Introduction**

- Thanks for your time
- Introductions
- We are very grateful for your participation in the pilot phase of our study. The goal of the study was to try out tools that we hoped would help people with pain move more throughout the day. The purpose of today’s conversation is to help our study team learn from your experience in the program. We would like to know more about what you found to be helpful and not helpful, and what you recommend that we do to improve the study.
- I consider you to be an expert consultant on your experience in the study and how we can best plan our next steps, and anything that you share is highly valuable to us.
- This interview will last no longer than an hour, and I would like to audio record our conversation so we can go back and document your feedback (ask for permission to record). If at any point you would like to take a break, skip a question, ask a question, or stop the interview, please just let me know.
- Do you have any questions for me before we start?

**MORPH Recruitment**

First, let’s talk about the very beginning of your involvement.

1. How did you hear about the study?
2. Why were you interested in participating?
3. In what ways did this program meet, exceed, or fail your expectations? Please explain.
   - What were your expectations?

**MORPH Components**

Next, let’s talk about your time with the MORPH program itself. We had several things we were interested in trying out in this program, so I’d like to chat with you about each of them.

1. The study team also sent you an ActivPAL activity monitor to wear throughout the study. Tell me about your experience setting up that device.
   - How easy or difficult was it to set up the ActivPAL device?
     - What would have made it easier?
     - Were you able to get assistance from the study team?
2. Tell me about your experience completing the daily surveys on your phone.
   - How easy or difficult did you find them to complete? Please elaborate.
3. We asked you to use the MORPH app throughout the program (this is different from the Fitbit App). How often did you try to use the app? Why?
4. Tell me about what you liked, or found useful about the app.
   - One of the goals of the app was to help you move more throughout the day. Do you feel as though the app helped you to better understand your physical activity levels? Why or why not?
   - Do you feel as though the app helped you achieve your physical activity goals? Why or why not?
5. What about the app did you not like, not understand, or not find useful?
6. What was the app missing? In other words, what did we fail to include that you would have found helpful?
   - Were there things that you wished were in the app that we did not have in the app?
7. We included a series of podcasts in the app. How often did you listen to the podcasts?
8. We included a series of cartoon videos in the app. How often did you watch the videos?
9. Tell me about your experience with using the scale at home.
   - Why was/wasn’t it useful to track your weight over time?
10. In addition to using the app, we asked you to meet in a group over video calling software each week What was your experience in the group meetings?
    - What did you like and dislike about the group meetings?
    - Do you feel like you meaningfully connected to others in your group?
      - Tell me more about that.
11. Tell me about your experience with the group leader.
    - Did you feel meaningfully connected to the group leader? Why/why not?
    - How clearly do you feel they explained each aspect of the program?
    - What are some things the leader could have done to help you feel supported in becoming more active?
    - If you had been the group leader, what would you have done differently?
12. Tell me about your experience with the student coaches.
    - Did you feel meaningfully connected to the student coaches? Why/why not?
    - In what ways did the student coaches support you to become more active?
    - What are some things the student coaches could have done to help you feel more supported?
    - In what ways do you think we can improve the training or preparation of student coaches?
13. In the future, we are considering including students as group leaders. What are your thoughts about this?
14. Which component(s) of the MORPH program do you think contributed the most to your experience? In other words, as we continue adapting the MORPH Program, which components should we keep in place? Why?

**Other Study Components**

**Over the course of the study, you were asked to complete questionnaires and assessments at home. Let’s talk for a minute about those.**

1. The study team sent you a kit with instructions and equipment to conduct a set of physical tests at home. Tell me about your experience with that.
   - How easy or difficult was it for you to complete these tests at home?
   - What could have made it easier?

**MORPH Program Overall**

**Let’s conclude by talking about the program overall**

1. Overall, what did you think about the MORPH program?

Since your involvement in MORPH –

1. Do you think differently about pain and how it relates to physical activity? Please explain.
2. Do you think MORPH has resulted in any long-term changes in your life? Please explain.
3. If you were to describe your experience with MORPH to someone who has never heard of it, how would you describe it?
4. Other than what we’ve already discussed, what additional suggestions for improving the MORPH program do you have for us? These changes could be related to the app, personnel or any of the study components.
5. And finally, is there anything else you’d like to tell us that we didn’t ask about?

Thank you for sharing your thoughts with me today!

**End Time: ________________**
